# Priming of plant resistance by natural compounds. Hexanoic acid as a model

**DOI:** 10.3389/fpls.2014.00488

**Published:** 2014-10-01

**Authors:** Paz Aranega-Bou, Maria de la O Leyva, Ivan Finiti, Pilar García-Agustín, Carmen González-Bosch

**Affiliations:** ^1^Departamento de Bioquímica y Biología Molecular, Universitat de Valencia, Instituto de Agroquímica y Tecnología de Alimentos, Consejo Superior de Investigaciones CientíficasValencia, Spain; ^2^Grupo de Bioquímica y Biotecnología, Área de Fisiología Vegetal, Departamento de Ciencias Agrarias y del Medio Natural, Escola Superior de Tecnologia i Ciències Experimentals, Universitat Jaume ICastellón, Spain

**Keywords:** priming, natural inducers, hexanoic acid, vitamins, oxidative stress, *Botrytis cinerea*

## Abstract

Some alternative control strategies of currently emerging plant diseases are based on the use of resistance inducers. This review highlights the recent advances made in the characterization of natural compounds that induce resistance by a priming mechanism. These include vitamins, chitosans, oligogalacturonides, volatile organic compounds, azelaic and pipecolic acid, among others. Overall, other than providing novel disease control strategies that meet environmental regulations, natural priming agents are valuable tools to help unravel the complex mechanisms underlying the induced resistance (IR) phenomenon. The data presented in this review reflect the novel contributions made from studying these natural plant inducers, with special emphasis placed on hexanoic acid (Hx), proposed herein as a model tool for this research field. Hx is a potent natural priming agent of proven efficiency in a wide range of host plants and pathogens. It can early activate broad-spectrum defenses by inducing callose deposition and the salicylic acid (SA) and jasmonic acid (JA) pathways. Later it can prime pathogen-specific responses according to the pathogen’s lifestyle. Interestingly, Hx primes redox-related genes to produce an anti-oxidant protective effect, which might be critical for limiting the infection of necrotrophs. Our Hx-IR findings also strongly suggest that it is an attractive tool for the molecular characterization of the plant alarmed state, with the added advantage of it being a natural compound.

## PRIMING PLANT DEFENSES

Plants are subjected to a variety of external factors that adversely affect their growth and development, and are often divided into biotic (insect herbivores and microbial pathogens) and abiotic (extreme temperature, inappropriate water supply, etc.) stresses. Adaptation to these environmental stresses is essential for survival and propagation (Rasmann et al., 2012). Among the plethora of defense strategies that plants have evolved, some are constitutive, but the majority are induced in response to stimuli, thus they are more specific (Frost et al., 2008). Recognition of different elicitors leads to the activation of diverse subsets of defense responses. Central regulatory hormones are salicylic acid (SA) and jasmonic acid (JA), although ethylene and abscisic acid (ABA), among others, also play key roles (Denance et al., 2013). For a plant, successfully tackling certain stress or a simultaneous group of stresses is a complex task, and responses largely overlap and can be interconnected positively and negatively (Ahmad et al., 2010). Induced resistance (IR) leads to various types of systemic resistance throughout the plant. IR is based on two general mechanisms: direct activation of defense responses in systemic tissue after local stimuli and priming, which implies activation of systemic responses, but only when the pathogen reaches these sites. The best characterized type of IR is systemic-acquired resistance (SAR), which is mostly dependent on SA, unlike the less understood JA-dependent defense (Conrath, 2009).

**FIGURE 1:**
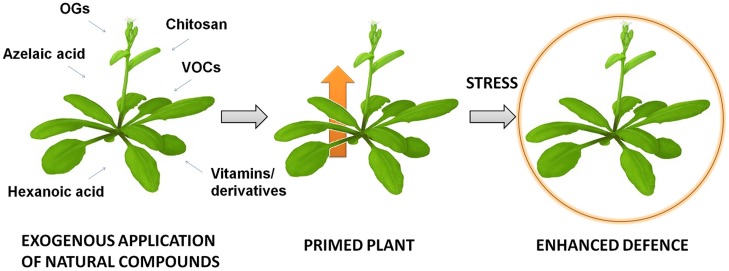
**Treatment with natural compounds increases plant protection against future stresses**.

Priming is a mechanism which leads to a physiological state that enables plants to respond more rapidly and/or more robustly after exposure to biotic or abiotic stress (**Figure 1**). The “primed” state has been related to increased, more efficient activation of the defense response and enhanced resistance to challenging stress (Conrath, 2009). This increased alertness correlates with no or minimal gene induction (Slaughter et al., 2012). The primed state results from the improved perception and/or amplification of defense response-inducing signals, rather than from the direct activation of these defense responses. Wide-ranging ways of inducing priming are known: infection by pathogens, colonization of roots by beneficial microbes, treatment with natural or synthetic chemicals, primary metabolism alteration and perception of certain volatile organic compounds (VOCs; Conrath et al., 2006). The molecular basis of priming has recently started to be unraveled, but is still poorly understood. Accumulation of both inactive mitogen-activated protein kinases (MAPKs) and transcription factors and certain epigenetic marks is best characterized (Conrath, 2011). The link between priming and epigenetic changes is further supported by the transgenerational priming phenomenon when the progeny of primed plants shows an enhanced defense response (Luna and Ton, 2012).

Induced resistance enables plants to activate the appropriate set of defenses in each situation to avoid misuse of resources and to minimize tradeoffs between defenses against different enemies. However, the time required to implement the response can mean that plants are subjected to considerable damage before the defense response acts. Priming probably evolved to compensate for this vulnerability, and to also allow plants to sense environmental cues and to promote a state of readiness to enable a quick, strong response upon pathogen attack (Frost et al., 2008). Priming is, indeed, the common theme underlying plant responses against both biotic and abiotic stress (Bruce et al., 2007). In this context, it is also worth noting that priming compounds do not tend to be highly specific, which can be an advantage or a disadvantage depending on the situation.

Over the years, a range of chemical treatments has proven capable of triggering IR, mostly through the priming mechanism. The first to be identified were synthetic SA analogs, such as 2,6-dichloroisonicotinic acid and its methyl ester (both referred to as INA), and benzo (1,2,3)thiadiazole-7-carbothioic acid *S*-methyl ester (BTH), which triggers SAR (Oostendorp et al., 2001; Conrath et al., 2002). A wide range of cellular responses has been reported to be potentiated by these compounds, including alterations in ion transport across the plasma membrane, synthesis and secretion of antimicrobial secondary metabolites (phytoalexins), cell wall phenolics and lignin-like polymers, and activation of various defense genes (Conrath, 2009). Non-protein amino acid β-aminobutyric acid (BABA) has received plenty of attention given its versatility, and its priming for different defense responses dependent on distinct hormones pathways and upon different challenging stresses (Conrath, 2009). This is remarkable because synthetic chemicals tend to prime SA-dependent immunity, as illustrated by the identification of priming-active compounds called imprimatins in synthetic library screening (Noutoshi et al., 2012).

## PRIMING BY NATURAL COMPOUNDS

Many natural compounds have been claimed to be plant growth promoters, plant activators or plant defense inducers, among other names. A large portion of them has already been related to priming, including oligosaccharides, glycosides, amides, vitamins, carboxylic acids, and aromatic compounds. In general, natural compounds tend to be better tolerated by plants than most of the synthetic compounds tested, but there is still concern about toxicity (Iriti et al., 2010; Noutoshi et al., 2012). Most mimic pathogen interaction by acting as (endogenous or exogenous) elicitors, and are able to induce or prime defense in a concentration-dependent fashion. However as Ahn et al. (2007) pointed out, the mode of action of priming agents is eventually determined by hosts and the stress challenging them. This makes it difficult to decipher the molecular bases underlying the priming mechanism. In addition, they usually show antimicrobial activities at higher concentrations than those required for priming. Overall they represent an active area of research in pest and disease management because of their versatility, their ability to prime JA-dependent defense and their general low toxicity, which allows better crop tolerance and fewer human health concerns usually associated with conventional strategies.

One group of successfully recently tested natural inducers in *Arabidopsis thaliana* consists in redox active compounds. Among them, thiamine (vitamin B1; Ahn et al., 2007), riboflavin (vitamin B2; Zhang et al., 2009), and quercetin (Jia et al., 2010) are all capable of inducing resistance by potentiating *Arabidopsis* sensitivity to *Pseudomonas syringae* elicitors. This leads to the activation of various plant defenses, such as the hypersensitive response, callose deposition and defense-related gene expression. The H_2_O_2_ burst seems to play a critical role as it acts as a signal to trigger the whole response. Although NPR1 is also required for priming by these compounds, the mechanism in this plant–pathogen interaction seems to act independently of classical defense pathways and is, perhaps, similar to the oxidative stress response. Recently, it has been demonstrated that thiamine can modulate the cellular redox status to protect *Arabidopsis* against *Sclerotinia sclerotiorum* at early stages of infection (Zhou et al., 2013). Early in the pathogenesis, thiamine can effectively alleviate the inhibition of host reactive oxygen species (ROS) generation by *Sclerotinia*-secreted oxalate. Thiamine can also induce cell wall fortifications with callose/lignin to prevent oxalate diffusion. Further reports in other plants are consistent with the central role of ROS, particularly H_2_O_2_ in vitamin-IR. The exogenous application of riboflavin primed bean, but not tomato plants, accelerates H_2_O_2_ generation after *Botrytis cinerea* infection. H_2_O_2_ is a signaling molecule involved in cell wall modification, gene expression regulation and cross-talk with various defense pathways (Azami-Sardooei et al., 2010). Riboflavin-IR also correlates with JA-dependent pathway activation by priming for enhanced lipoxygenase (LOX) activity. LOX enzymes are involved in the first steps of the octadecanoid pathway, which leads to oxypilin synthesis, like JA, and renders various intermediate compounds with defense implications (Azami-Sardooei et al., 2010; Taheri and Tarighi, 2010). The up-regulation of the phenylalanine ammonia-lyase (*PAL*) gene and peroxidase (*cprx1*) genes implicated in the phenolic metabolism has also been observed in sugar beet (both) and rice (only *PAL*) after riboflavin application and challenging with *Rhizoctonia solani*. Phenolics play a role in cell wall fortification, and also show antimicrobial and antioxidant activity (Taheri and Tarighi, 2010, 2011).

Para-aminobenzoic acid (PABA) is a cyclic amino acid belonging to the vitamin B group. Field experiments have proven that it is capable of enhancing resistance against Cucumber mosaic virus and *Xanthomonas axonopodis* by inducing SAR, while simultaneously improving plant yield (Song et al., 2013). This contrasts with BTH which, in the same study, reduced disease severity, but produced shoot length shortening and significant fruit weight reduction when compared to PABA and control treatments.

Menadione sodium bisulfite (MSB) is a vitamin K3 derivative known to be a growth regulator (Rama Rao et al., 1985). Borges et al. (2003a) found that MSB protects rape plants (*Brassica napus*) from the fungus *Leptosphaeria maculans* by stimulating ROS production, but without inducing *PR1*. Other authors have shown that MSB has a systemic effect (Liu et al., 2007) and reported H_2_O_2_ production induced by the compound through gene induction (Benitez et al., 2005). Borges et al. (2003b, 2004) also demonstrated that MSB protects banana from Panama disease caused by *Fusarium oxysporum* and that MSB primes phytoalexin accumulation. Later on, these authors demonstrated that MSB induces *Arabidopsis* resistance against *P. syringae* via a priming mechanism as MSB induces only ROS and *PR1* accumulation on post-inoculation day 3 (Borges et al., 2009). In their study, the authors analyzed gene expression profiling after menadione treatment by microarray technology. MSB produced a unique molecular footprint, but most up-regulated genes have been previously connected to stress. Furthermore, the G-box in their promoters was over-represented, and, interestingly, other up-regulated genes coded for transcription factors, including the putative regulators of the G-box (Borges et al., 2009). It is remarkable that a menadione derivative (Param-A) has been commercially launched to induce resistance against Panama disease in bananas because when this derivative is sprayed, can significantly reduce disease occurrence and delay symptom appearance in the field (Fernández-Falcón et al., 2009).

Chitosan is a polymeric deacetylated derivative of chitin that is naturally present in some fungi cell walls, and has various deacetylation degrees and molecular weights. Although it performs several antimicrobial activities, its main contribution to reduce plant disease is to enhance plant defenses (El Hadrami et al., 2010). Chitosan has also been reported to improve growth and yield (Reddy et al., 1999; Kim et al., 2005; Cho et al., 2008). It is a potent general elicitor of proven efficiency in a wide range of experiments with different host plants and pathogens (Iriti et al., 2010). Iriti and Faoro (2009) pointed out that chitosan can directly activate systemic resistance or can prime the plant for a more efficient defense response upon challenge, depending on dose, by considering the different cytotoxicity thresholds for each chitosan derivative and plant. The diverse mechanisms of action of chitosan have been studied, which include oxygen-species scavenging and antioxidant activities, as well as octadecanoid pathway activation (reviewed in El Hadrami et al., 2010). Despite these studies however, experiments which specifically address the role of priming in the complex chitosan-plant interaction framework are still scarce.

There is evidence to support that the wound signal from the local attack site is transmitted to systemic undamaged regions, where priming or the direct induction of defense responses takes place. Signal transmission can occur either internally, probably through the phloem and xylem, or externally via VOCs (Frost et al., 2008). In the internal signaling mechanism, pathogen-induced damage in the plant cell wall can be the starting point. Cell wall degrading enzymes, such as Endo-1,4-β-glucanases, have also been found to be implicated in IR defense pathways (Flors et al., 2007; Cantu et al., 2008; Finiti et al., 2013). It is widely accepted that the plant cell wall is a dynamic functional structure involved in several plant processes, including response to stress (Huckelhoven, 2007). The elicitors released from it during pathogen infection contribute to basal resistance against fungal pathogens via a signaling pathway, which is also activated by pathogen-associated molecular pattern molecules. However, the actual components and pathways remain largely unidentified (Osorio et al., 2008).

Oligogalacturonides (OGs) are plant cell wall pectin-derived oligosaccharides which consist in linear chains of α-(1-4)-linked D-galacturonic acid with a degree of polymerization between 10 and 25, which can be methyl-esterified or acetylated depending on the source plant. They are considered endogenous elicitors, and the degree of methylation and acetylation has been found to affect the activation of defense responses (Osorio et al., 2008; Randoux et al., 2010). OG treatment has been reported to induce a range of defense responses, like accumulation of phytoalexins, β-1,3-glucanase and chitinase, or generation of ROS by triggering nitric oxide (NO) production (Rasul et al., 2012). Interestingly, some evidence indicates the involvement of OGs signaling in the octadecanoid pathway, whereby LOX activities are enhanced (Randoux et al., 2010). Exogenous treatments with OGs protect grapevine leaves against necrotrophic pathogen *Botrytis cinerea* infection in a dose-dependent manner (Aziz et al., 2004). In *Arabidopsis*, OGs increase resistance to *Botrytis cinerea* independently of JA-, SA-, and ethylene (ET)-mediated signaling. A microarray analysis has shown that about 50% of the genes regulated by OGs display a similar change of expression during *Botrytis cinerea* infection (Ferrari et al., 2007).

Azelaic acid (AA) has been suggested to be a phloem-mobile signal that primes SA-induced defenses (Jung et al., 2009; Shah, 2009). The AA biosynthesis pathway is largely unknown, although recent evidence indicates that it is a derivative of oleic acid or its desaturated derivatives, linoleic and linolenic acids (Yu et al., 2013). Lipid peroxidation has been proposed as being responsible for AA formation and can proceed by LOX activities or the fragmentation pathway triggered by ROS. In addition, other stress-signaling molecules are generated (Zoeller et al., 2012). AA primes plants for more rapid SA accumulation by inducing glycerol-3-phosphate (G3P) biosynthesis (Yu et al., 2013). G3P levels have been proposed to modulate primary and secondary metabolic pathways, and to contribute to major physiological responses in defense (Chanda et al., 2008). So both AA and G3P seem to be implicated with phytohormones SA and JA. A synergy between AA and dehydroabetinal (DA) signaling has been suggested. DA is an abietane diterpenoid released upon wounding that is induced locally by insect infestation. There is evidence to suggest that it translocates rapidly through the plant and acts as a SAR inducer (Chaturvedi et al., 2012). Further research is required to address the implication of priming in this interaction and in DA-IR.

Along with AA, G3P and DA, pipecolic acid (L-Pip) a Lys-derived non-protein amino acid has been recently implicated as pivotal regulator of SAR, and possibly as the long-distance phloem-mobile SAR signal compound (Navarova et al., 2012; Shah and Zeier, 2013). Amino acids (aa) metabolism plays an increasingly wide range of roles in plant immunity. For example, proline metabolism has been related to oxidative burst and to the establishment of the hypersensitive response; branched chain aa catabolism mediates the cross-talk between SA and JA defenses; acetylated aa form phytohormone-aa conjugates (Zeier, 2013). Apart from these, L-Pip has been identified as a central node in SAR. L-Pip acts as an endogenous mediator of defense amplification in SAR, and also in BABA-IR. L-pip activates SA biosynthesis and its own biosynthesis via a positive feedback loop to thus orchestrate the whole SAR response (Navarova et al., 2012). Interestingly, the exogenous application of L-Pip primes *Arabidopsis* plants for more rapid SA biosynthesis, phytoalexin camalexin accumulation and defense gene expression (Navarova et al., 2012). It also primes tobacco plants for quicker SA biosynthesis and nicotine accumulation (Vogel-Adghough et al., 2013).

Another carboxylic acid with demonstrated inducer activity is hexanoic acid (Hx; Vicedo et al., 2009). We focus on this natural compound as a model for priming by natural compounds in the section below.

Volatile organic compounds play key roles in plant–plant communication as they act as airborne signals by enhancing disease resistance in the plant itself and in neighboring plants, and by also attracting parasitic or predatory insects, these being the enemies of attacking herbivores (Arimura et al., 2010). This multifunctional role makes them desirable for sustainable pest control strategies (Oluwafemi et al., 2013). A subset of VOCs emitted in response to insect attack is called herbivore-induced plant volatiles (HIPVs). Some HIPVs are known to be green leafy volatiles (GLVs), and they usually form from linolenic and linoleic acids as one of the oxypilin pathway branches. GLVs are first emitted upon wounding or herbivore attack (Hirao et al., 2012). They induce a wide range of defense reactions, probably almost entirely based on priming under field conditions, and they also display antibacterial and antifungical activities. The defense reactions linked to VOCs effects include enhanced phytoalexins secretion, incorporation of hydroxycinnamic acid esters and “lignin-like” polymers into the cell wall, enhanced oxidative burst, augmented induction of defense genes, emission of aromatic compounds and quicker trypsin inhibitors production (Conrath, 2009). GLVs prime plants for a more robust defense response by increasing the total VOCs emission and endogenous JA content after detecting an elicitor (Engelberth et al., 2004; Kishimoto et al., 2005). They also seem to increase sensitivity to methyl jasmonate (MeJA), the methyl ester of JA (Hirao et al., 2012). Interactive effects of different VOCs have been described for (Z)-3-hexen-1-ol and ethylene, although ethylene does not seem to have any effect on its own, which implies that it is worth investigating the role of each VOC and its interactive effects (Frost et al., 2008). (Z)-3-hexen-1-ol apparently plays a twofold role by priming and modulating the behavior of herbivorous insects (Wei and Kang, 2011).

Cis-Jasmone (CJ) is a highly volatile compound product of further catabolism of JA, which is known to induce the release of defense VOCs that attract predatory/parasitic insects (Birkett et al., 2000). A transcriptomic analysis has shown that CJ treatment triggers the up-regulation of a unique subset of genes, including cytochrome P450 family members. It has also been indicated that the CJ-induced expression acts independently of COI1, which is the F-box protein mediating the MeJA-induced gene expression (Bruce et al., 2008). Recently, this oxypilin has been demonstrated to enhance defense by priming plants for the quicker induction of VOCs release. The same study has suggested that CJ primes JA-induced pathways, such as the sesquiterpene synthase gene expression, despite its independent signaling roles (Oluwafemi et al., 2013).

## HEXANOIC ACID PRIMING AGENT

**FIGURE 2:**
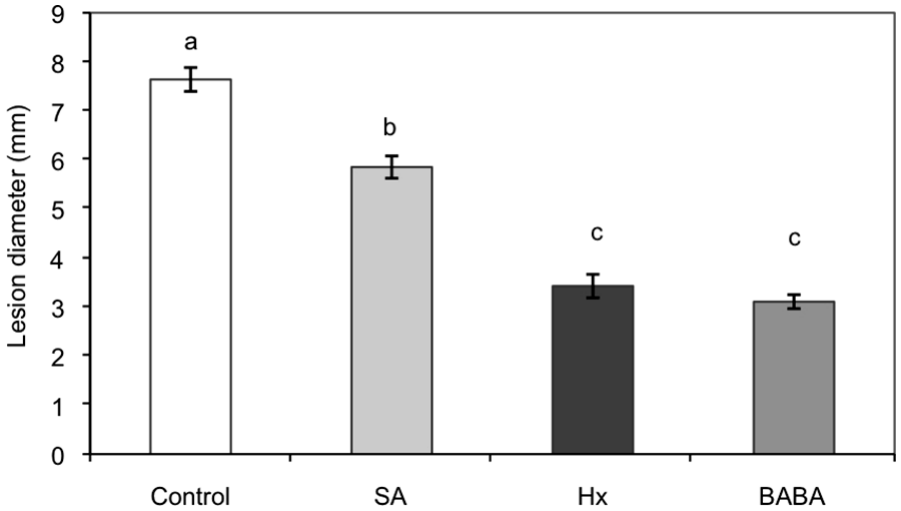
**Root treatment with hexanoic acid protects tomato plants against *Botrytis cinerea* infection.** Four-week-old tomato plants (cv. Ailsa Craig) were treated with 0.6 mM hexanoic acid, 0.5 mM SA, and 0.5 mM BABA under hydroponic conditions. Control plants were treated with water. Lesion diameter was measured at 72 h after inoculation. Data show the lesion diameter (mm) ± SE (*n* = 20). Different letters represent statistically significant differences (*p* < 0.05; least significant difference test). Figure modified from Vicedo et al. (2009).

We have previously demonstrated that root treatment with natural 6C monocarboxylic acid Hx protects tomato plants against necrotrophic fungi *Botrytis cinerea* (Leyva et al., 2008; Vicedo et al., 2009). Root treatment of 4-week-old plants with Hx at concentrations below 1 mM for 48 h prior to infection significantly reduced the incidence of the disease as other well known natural (SA) and non-natural (BABA) inducers did (**Figure 2**; Vicedo et al., 2009). At these concentrations Hx had no antimicrobial effect on *Botrytis cinerea* and shorter conditioning times were not sufficient to protect the plant against this pathogen, which strongly supports the inducer effect of this treatment. In addition, Hx did not accumulate in the aerial part of the plant, suggesting that protection might result from specific interactions with plant defense systems. Hexanoic treatment induced callose accumulation upon *Botrytis cinerea* infection. Cell wall fortification by callose deposition is a key component of resistance induced by chemical inducers like BABA or BTH (Kohler et al., 2002). Hx treatment also increased caffeic acid levels after fungal infection, which further supports the role of a reinforced cell wall in Hx-IR. Callose priming forms part of Hx-IR in different cultivars against *Botrytis cinerea* (Ailsa Craig, Moneymaker and Rheinlands Ruhm). However, the plants from Castlemart are protected by Hx in the absence of callose priming (Vicedo et al., 2009). This result indicates that additional mechanisms are involved in Hx-IR.

Bioactive signal jasmonoyl-isoleucine (JA-Ile; Chico et al., 2008) increased sharply in Hx-primed tomato plants after *Botrytis* inoculation. In addition, Hx-IR was blocked in the JA-insensitive mutant *jai1*, a *coi1* homolog (Vicedo et al., 2009), which is impaired in receptor COI1 (Yan et al., 2009). Oxylipin12-oxo-phytodienoic acid (OPDA) was also primed by Hx treatment after fungal infection (Vicedo et al., 2009). OPDA, a precursor of JA, is partly a regulator of plant defenses in a JA-independent manner and is also active against microorganisms, including *Botrytis cinerea* (Stintzi et al., 2001; Prost et al., 2005). The increase in JA observed in water-treated plants upon infection was not detected in Hx-treated plants, which suggests that this hormone recirculates into its conjugated forms, like JA-Ile (Vicedo et al., 2009). The analysis of tomato genes induced in response to *Botrytis* showed that, consistently with metabolic changes, Hx pre-treatment significantly primes *LoxD*, a LOX involved in the oxylipins pathway leading to OPDA and JA synthesis (Cohn and Martin, 2005; Flors et al., 2007).

Abscisic acid-deficient mutant *flacca* (flc) was also impaired in Hx-IR in three different backgrounds (Vicedo et al., 2009) and correlated with the absence of callose priming upon infection. Therefore, ABA can act as a positive regulator of Hx-IR by enhancing callose deposition, as previously reported for BABA-IR in *Arabidopsis* (Ton and Mauch-Mani, 2004).

## HEXANOIC ACID IS A BROAD-SPECTRUM NATURAL INDUCER

Hexanoic acid treatment has also been found to protect *Arabidopsis* plants against *Botrytis cinerea* (Kravchuk et al., 2011). In this case, Hx-IR has also been associated with changes in the JA-signaling pathway upon infection. The JA and ET defense-response marker gene *PDF1.2* (Penninckx et al., 1996), the JA-regulated hevein-like protein gene *PR4* (Van Damme et al., 1999) and the specific JA-inducible marker gene *VSP1* (Norman-Setterblad et al., 2000) were primed in Hx-treated plants upon *Botrytis* infection. The JA and OPDA levels were induced at early stages in Hx-treated plants upon infection, as previously observed in tomato. Accordingly, JA-impaired mutant *jar1* and *jin1-2* were unable to display Hx-IR. JAR1 encodes an enzyme that conjugates JA with Isoleucine (Staswick et al., 2002), while JASMONATE-INSENSITIVE1 (JAI1/JIN1) encodes AtMYC2, which is a nuclear-localized transcription factor whose expression is rapidly up-regulated by JA content (Lorenzo et al., 2004). Thus, the metabolic switch for hexanoic must act upstream of both genes. Further analyses have demonstrated that the plants impaired in the ET, SA, and ABA pathways show intact protection by Hx upon *Botrytis cinerea* infection. Accordingly, no significant changes in SA marker gene *PR1* and in the SA or ABA hormone balance were observed in infected and treated plants. The *eds1-1* mutant (Zhou et al., 1998; Falk et al., 1999) was unable to display Hx-IR. EDS1 (ENHANCED DISEASE SUSCEPTIBILITY1) is a nucleocytoplasmic lipase-like involved in plant defense signal transducing (Brodersen et al., 2006; Heidrich et al., 2011). Future research will clarify the possible role of this key regulator in Hx-IR.

Callose accumulation is also primed in Hx-treated *Arabidopsis* plants upon infection. However, it is not essential to express Hx-IR in *Arabidopsis* since the *pmr4-1* mutant (Powdery Mildew Resistant 4-1; Nishimura et al., 2003) is protected by Hx, despite it being unable to synthesize callose. This differs from Hx-IR in tomato, where callose plays an important role in resistance against *Botrytis*. The main function of callose is to act as a physical barrier against pathogens, but it may also reduce the permeability of the toxins secreted by pathogens, and of other molecules. This scenario has been suggested in *Arabidopsis* plants primed by thiamine against *Sclerotinia sclerotiorum* (Zhou et al., 2013). Hx-IR against *Botrytis cinerea* also seems to be independent of ABA production in *Arabidopsis* since the mutant partially impaired in ABA synthesis *npq2-1* (Niyogi et al., 1998) was fully protected by Hx (Kravchuk et al., 2011). All these results indicate that Hx priming against *Botrytis cinerea* is based on JA- and other oxylipin-related defenses in both tomato and *Arabidopsis* by activating additional responses in each background.

Hexanoic acid has also been seen to increase resistance against necrotrophs *Alternaria brassicicola* in *Arabidopsis* (Kravchuk et al., 2011) and *Alternaria alternata* in Fortune mandarin (Llorens et al., 2013). In this case, both JA-signaling and callose priming were required for Hx-IR. Furthermore, a more rapid accumulation of ABA was observed, which could act as a positive regulator of callose deposition, as described in tomato, thus reinforcing the fact that both enhanced physical barriers and the JA-signaling pathway are involved in Hx-IR against necrotrophic pathogens.

Hexanoic acid has been reported to protect tomato plants against hemibiotrophic bacterium *P. syringae* pv tomato DC3000 (Vicedo et al., 2009). In this case, Hx-IR seems to counteract the negative effect of the pathogen coronatine (COR) and JA-Ile on the SA pathway (Scalschi et al., 2013). Hx treatment reduced JA-Ile content upon infection at the expense of an increased expression of jasmonic acid carboxyl methyltransferase (JMT) and of SA marker genes *PR1* and *PR5,* which indicates a boost in this signaling pathway. Interestingly, Hx treatment prompted OPDA accumulation, as seen in tomato and in *Arabidopsis* against *Botrytis cinerea*, suggesting that this molecule might play a role *per se* in Hx-IR. Hence the obtained results support a positive relationship between the SA and the JA pathways in Hx-primed plants. Hx also seems to inhibit stomatal opening in tomato plants in the presence of COR, which implies that this treatment suppresses pathogen effector action to prevent bacterial entry into the mesophyll (Scalschi et al., 2013). Therefore, Hx induces plant responses in different host plants and against pathogens with distinct lifestyles through a common strategy based on the priming of OPDA accumulation and JA-signaling by diverting it toward the accumulation of different JA-conjugates that depend on the attacking pathogen.

## HEXANOIC ACID REGULATES AND PRIMES *Botrytis*-SPECIFIC AND NON-SPECIFIC GENES

We recently analyzed the gene expression profile of *Botrytis*-infected tomato plants 24 hpi by microarray analysis (Finiti et al., 2014). The results indicated that tomato plants respond early to *Botryti*s inoculation by activating a large set of genes, which are mainly related with the biotic stress response, and interestingly with the oxidative stress response. The most induced genes were proteinase inhibitors, defense genes (especially fungus and chitin activated-genes), transcriptional factors, and signaling and hormone-related genes. Oxylipins-, ethylene-, and auxin-related genes were induced, which corroborate the involvement of these pathways in the early response to *Botrytis*. Remarkably a set of redox-related genes was also induced, which evidences the involvement of oxidative stress mechanisms in this plant–pathogen interaction.

The microarray gene expression profile of Hx-treated plants revealed the induction and priming of many *Botrytis*-induced genes (Finiti et al., 2014). This means that Hx preventively activates these genes, thus preparing plants for an alarmed state, which would facilitate a quicker, better response against pathogen attack. Hx is also capable of priming and enhancing the expression of many of those genes after fungus inoculation. This confers increased resistance to treated plants without wasting resources until infection occurs. It is noteworthy that Hx activated a set of genes, which was not induced by the fungus at 24 hpi. These specific Hx early induced genes may prove advantageous for treated plants, and could be further studied as targets of new preventive defense strategies (Boyd et al., 2013).

The microarray technique revealed a high induction in infected Hx-treated plants of the genes encoding for proteinase inhibitors responsive to JA, wounding and insect feeding (Farmer et al., 1992). This evidences the relevance of the JA-pathway in Hx-IR, and how proteinase inhibitors might play a key role in the tomato-*Botrytis* interaction. Genes coding for proteinase inhibitors could also represent new targets for treatments and genetic engineering to increase plant resistance.

The genes involved in the oxylipins pathway, such as *LoxD*, *DES* (divinyl ether synthase) and *Dox1* aplha-dioxygenase, were induced and primed by Hx (Finiti et al., 2014), thus supporting the priming of this metabolic pathway as part of Hx-IR (Vicedo et al., 2009). Notably, the 1-aminocyclopropane-1-carboxylic acid (ACC) oxidase gene was also primed by Hx. According to Diaz et al. (2002), early ET synthesis activation prior to pathogen attack can increase plant resistance against *Botrytis cinerea.* Hence, the pre-activation and boost of the *ACC* oxidase gene expression found in treated plants likely contributes to increased resistance. SA-responsive defense genes, like *PR1a* and *endochitinase 3,* were also induced in treated plants. This shows the complex effect of Hx priming on the hormonal balance, with a likely positive effect on both the JA and SA pathways, traditionally considered antagonist, as previously observed in Hx-IR against *Pst* (Scalschi et al., 2013).

Many genes encoding for WRKY family members, which modulate the defense response, were induced and primed in Hx-treated plants (Finiti et al., 2014). Huang et al. (2012) reported the involvement of SlWRKYs (*Solanum lycopersicum* WRKYs) in responses to different abiotic and biotic stresses, including *Botrytis cinerea* infection. Among the Hx-induced WRKYs, there are orthologs of *Arabidopsis WRKY18*, *WRKY33*, *WRKY40*, *WRKY53* and *WRKY75*, which form part of the *Arabidopsis* defense response, especially against *Botrytis cinerea* (AbuQamar et al., 2006; Pandey et al., 2010; Birkenbihl et al., 2012). Hx’s capability of acting on these regulatory multi-response factors probably contributes considerably to its broad-spectrum IR.

It is particularly noteworthy that Jaskiewicz et al. (2011) have recently reported how *WRKY53* is a specific target of BTH priming in *Arabidopsis*, and that it might be considered a priming marker gene. According to the above-cited authors, priming occurs through changes in histone acetylation before pathogen inoculation. Then the pre-acetylated gene is induced more quickly upon pathogen recognition, leading to higher expression levels and a better defense response. Hence, the over-induction of *WRKY53* observed in Hx-treated plants upon infection supports Hx playing a priming agent role, and being capable of preparing the plant in a silent mode without wasting too much energy until a pathogen is detected.

## Hx TREATMENT ALLEVIATES OXIDATIVE STRESS

Oxidative burst and ROS accumulation are critical factors in plant responses to *Botrytis* infection (Heller and Tudzynski, 2011), but the contribution of these factors to plant defense is complex because *Botrytis* stimulates ROS production to its own benefit (Temme and Tudzynski, 2009). Microarray data have indicated that the response of Hx-treated tomato plants is similar to the response of those inoculated with *Botrytis,* which reveals the activation of many redox status-related genes (Finiti et al., 2014). Several of the genes induced by *Botrytis* are over-induced and primed in Hx-treated plants, including peroxidase, glutathione reductase, NADPH quinone reductase, and several glutathione *S*-transferases (GSTs). Other genes, like GST and glutaredoxin, are induced only in treated plants and represent specific targets of the inducer treatment. The early boosting of detoxifying and redox-balance-related genes achieved by Hx supports the direct effect of this inducer on the control of these processes. The analysis of oxidative stress markers confirmed that Hx treatment protects plants by providing a less oxidized cellular environment after infections (Finiti et al., 2014). Superoxide ion (O_2_-) and peroxide hydrogen (H_2_O_2_) accumulation reduced and was more restricted around the infection site. The ascorbate and glutathione reduced/oxidized ratios rose in treated plants at 72 hpi, while the activities of glutathione reductase and catalase remained closer to those of healthy plants. No changes were detected in Hx-treated, but not-infected, plants. Therefore, Hx primes the transcription of the genes controlling the redox metabolism, which is fully activated and shown only after pathogen recognition (Finiti et al., 2014), just as the priming definition establishes (Conrath et al., 2002). Hence Hx treatment can limit oxidative stress in infected plants by damping the fluctuations of the redox equilibrium and preventing its harmful effects in later infection steps. Interestingly in *Arabidopsis*, wound-IR against *Botrytis cinerea* also requires glutathione and the priming of the gene encoding *GST1* in leaves inoculated with the fungus (Chassot et al., 2008). Studies carried out with the tomato ABA-deficient mutant *sitiens* have also revealed that the timely hyperinduction of H_2_O_2_-dependent defenses on the epidermal cell wall can effectively block early *Botrytis cinerea* development (Asselbergh et al., 2007), which further demonstrates the importance of oxidative stress in this plant–pathogen interaction.

As previously mentioned, other natural priming agents which promotes defense response like thiamine, riboflavin, MSB, VOCs, OGs, and chitosan also affect the oxidative balance contributing to reduce the symptoms and damages associated to biotic stresses (**Table 1**). Thiamine can modulate the cellular redox status by activating the NADPH oxidase and promoting early ROS generation, which confers resistance against *Sclerotinia sclerotiorum* in *Arabidopsis* (Zhou et al., 2013). Riboflavin promotes the H_2_O_2_ burst independently of the known hormonal pathways in *Arabidopsis*, suggesting a distinct signaling process for this priming compound. MSB is a ROS generator too, but it also induces detoxification genes like several GSTs and ABC transporters that may scavenge toxic compounds generated during oxidative stress (Borges et al., 2009). In recent years attention has been directed toward the antioxidant activity of chitosan. It promotes ROS generation mainly through the plasma membrane NADPH oxidase, inducing the hypersensitive response and programmed cell death. However, the water-soluble chitosan is an excellent scavenger of hydroxyl radicals, H_2_O_2_ and anion superoxide, revealing the diverse properties of this compound (El Hadrami et al., 2010).

**Table 1:** Natural inducers and their effects on plant defensive mechanisms reported in this work.

Inducer	Plant	Pathogen	SA*	JA* oxilipins	ET*	SAR	Defense effectors	Cell wall tightening	Oxidative balance	Reference
Hexanoic acid	*Tomato*	*Botrytis cinerea*	+	+	+	Nd	+	+	+	Vicedo et al. (2009), Finiti et al. (2013)
	*Arabidopsis*	*Botrytis cinerea*	Nd	+	Nd	Nd	+	+	Nd	Kravchuk et al. (2011)
	*Tomato*	*P. syringae*	Nd	+	Nd	Nd	+	+	Nd	Scalschi et al. (2013)
Thiamine	*Rice, Arabidopsis*	Fungal, bacterial, viral infections	+	-	-	+	-	+	+	Ahn et al. (2007)
Riboflavin	*Arabidopsis*	*P. syringae*	-	-	-	+	+	+	+	Zhang et al. (2009)
	*Tomato*	*Botrytis cinerea*	Nd	+	-	+	Nd	Nd	-	Azami-Sardooei et al. (2010)
PABA	*Pepper*	CMV, *Xanthomonas*	+	-	Nd	+	+	Nd	Nd	Song et al. (2013)
MSB (k3)	*Arabidopsis*	*P. syringae*	Nd	Nd	Nd	-	+	Nd	+	Borges et al. (2009)
VOCs	*Maize, Bean, Arabidopsis*	Insects	Nd	+	+	Nd	+	+	+	Frost et al. (2008)
OGs	*Arabidopsis*	*Botrytis cinerea*	-	-	-	-	+	Nd	+	Ferrari et al. (2007)
Azelaic acid	*Arabidopsis*	*P. syringae*	+	-	-	+	+	Nd	Nd	Jung et al. (2009)
Pipecolic acid	*Arabidopsis*	*P. syringae*	+	Nd	Nd	+	+	Nd	Nd	Vogel-Adghough et al. (2013)
Chitosan	*Soybean, tomato, maize*	Fungal, bacterial, viral infections	Nd	+	Nd	Nd	+	+	+	El Hadrami et al. (2010)
		*Colletotrichum sp*	Nd	+	Nd	Nd	+	+	+	
		*Xanthomonas*	Nd	+	Nd	Nd	+	+	+	
	*Broccoli*	*P. fluorescens*	Nd	+	Nd	Nd	+	+	+	Li et al. (2010)

Abbreviations: (+) activates, (-) does not activate, (Nd) not determined.

*SA, JA, and ET are figured as dependency (+) of the pathway, or not (-).

Priming agents that increase abiotic stress tolerance have been also associated with oxidative stress control. Sodium hydro-sulfide (NaHS) protects plants from salinity and non-ionic osmotic stress by altering the redox machinery in a similar way than Hx (Christou et al., 2013). Treatment with NaHS maintains low ROS concentration in stressed strawberry plants activating enzymatic antioxidants such as superoxide dismutases, catalases and ascorbate peroxidases. It also increases the ascorbate and glutathione redox states and induces the expression of key genes for ascorbate and glutathione biosynthesis.

In conclusion, microarray data of Hx-treated plants have revealed the induction of many genes that help characterize the Hx priming effect, especially those related with defense, the signaling network and oxidative stress control, which are over-induced in Hx-treated plants upon fungal infection. The activation and priming of different defense genes responding to the SA and JA pathways match the broad-spectrum action of this natural inducer. This agrees with the present conception that the effectiveness of the plant response against biotrophic and necrotrophic pathogens is much more complex than the classical dichotomy between the SA and JA pathways antagonism. Finally, Hx priming of redox-related genes produces an anti-oxidant protective effect, which might be critical for limiting necrotroph infection. These findings back the importance of controlling oxidative stress to improve plant protection against different pathogens, and suggest that this natural inducer is an attractive tool to further study this topic.

## ANTIMICROBIAL ACTIVITY OF Hx

Some natural compounds, which act as inducers, may also have a direct antimicrobial effect under certain conditions. Among these we find chitosan (Ben-Shalom et al., 2003) and some plant volatile compounds (He et al., 2006). This is the case of Hx as it also inhibits *Botrytis cinerea* growth at higher concentrations than those which allow the priming of plant defenses (Leyva et al., 2008). Hx blocks spore germination at a very early stage, prevents germ-tube development and also inhibits *in vitro* mycelia growth of germinated spores. Once again, this reflects the remarkable versatility of this natural compound to act on the plant and pathogen in a concentration-dependent manner.

The characterization of the mechanisms underlying the antimicrobial effect of Hx shows a retraction of the cytoplasm in treated spores, as previously demonstrated for other natural compounds with antifungal properties against *Botrytis cinerea,* such as resveratrol (Gonzalez Ureña et al., 2003) and synthetic compound adipic acid monoethyl ester (AAME; Vicedo et al., 2006). Hx treatment of previously germinated spores has altered fungal membrane permeability by producing a phosphate efflux with no lytic activity. A similar effect has been observed for not only several diterpenoids with antifungal activity against *Botrytis cinerea* (Cotoras et al., 2004), but also for a series of aliphatic (2 E)-alkenals, from C5 to C14, characterized as antimicrobial agents (Kubo et al., 1995). In addition, Hx treatment has been reported to increase the levels of spermine, spermidine, putrescine, and cadaverine in *Botrytis cinerea* mycelia. Polyamine metabolism is a target of other antifungal compounds (Walters, 1995) and plays a main role in programmed cell death (Walters, 2000).

**FIGURE 3:**
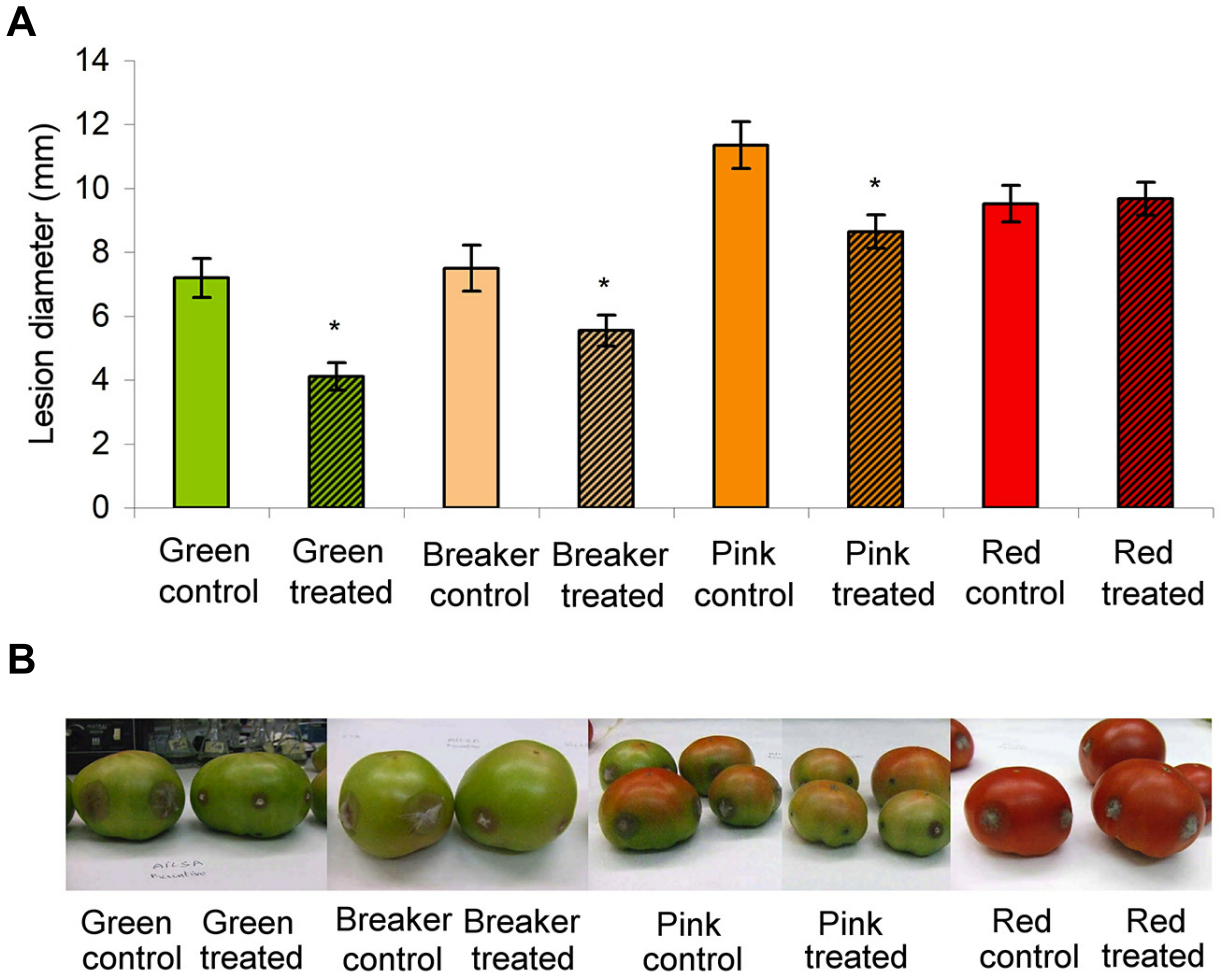
**Treatment with hexanoic acid reduces the disease symptoms in tomato fruits infected with *Botrytis cinerea*.** Tomato fruits (cv. Ailsa Craig) harvested at different ripening stages were wounded and inoculated with 5 μL of a *Botrytis cinerea* conidia suspension in each wound. 24 h later, when the first symptoms of infection are already visible, twenty fruits were sprayed with Hx 20 mM. Control fruits were sprayed with sterile water. Three days after treatment fruits were scored for symptoms by measuring the lesion diameter. Statistically significant differences are indicated with (*p-value < 0.05). Representative images of the infected fruits are also provided. Figure from Leyva Pérez (2008).

The twin effect of Hx on both germination and mycelia growth has been observed only in highly efficient synthetic fungicides, such as phenylpyrroles and hydroxyanilides (Rosslenbroich and Stuebler, 2000). This twin effect was also confirmed *in planta*. Spraying Hx at fungicidal concentrations (16 mM) on tomato plants prior to fungal inoculation reduces the diameter of necrosis by ∼60%. Application on previously infected plants further reduces necrotic expansion by around 30% (Leyva et al., 2008). We have also observed this preventive and curative effect on mature green fruits and its curative effect on fruits in different ripening stages (**Figure 3**; Leyva Pérez, 2008). The antifungal properties of Hx have also been demonstrated in Micro-Tom tomato plants (Leyva Pérez, 2008) by making the most of its susceptibility to a wide range of pathogens (Takahashi et al., 2005) and the possibility of infecting fruit-producing plants with *Botrytis cinerea* under laboratory-controlled conditions. Spraying Hx at fungicidal concentrations on 2-month-old plants prior to fungal inoculation reduces the diameter of necrosis by ∼15%. Application of Hx on previously infected plants further reduces necrotic expansion by around 60% (Leyva Pérez, 2008). These treatments have no phytotoxic effects and demonstrate the ability of Hx to prevent and reduce *Botrytis* infection in tomato plants and fruits. This feature makes Hx a good candidate to protect tomato crops and for post-harvest application at either fungicide or inducer concentrations.

## CONCLUSION AND FUTURE PERSPECTIVES

The study into natural plant inducers has helped unravel the complex mechanisms underlying the IR phenomenon (**Table 1**).

As this review shows, Hx-IR shares the protection strategies and mechanisms promoted by several vitamin treatments, like JA dependent pathway activation. Hx can modulate the cellular redox status to protect tomato plants against *Botrytis cinerea* in an early infection stage, as demonstrated for thiamine against *Sclerotinia sclerotiorum* in *Arabidopsis*. In addition, both natural priming agents induce cell wall fortifications with callose. However, the fact that Hx-IR primes some genes like GST and glutaredoxin, which are not early induced by *Botrytis* in tomato, indicates that these genes can be the direct targets of this natural inducer. It is noteworthy that one of the most highly induced genes by MSB, a water-soluble derivative of vitamin K3, encodes for a GST, which is highly induced by H_2_O_2_ and NO treatments. Hence, a common priming mechanism can relay the more efficiently toxic compounds generated during oxidative stress in scavenging.

Another interesting contribution that stems from studying resistance induced by natural compounds, including Hx, is the evidence found that oxylipins are involved in the priming mechanism in a JA-independent manner. Hx-IR correlates with OPDA accumulation in all the studied pathosystems. It is stressed that the transcriptome analysis of the *Arabidopsis* response to OPDA revealed that 17% of induced genes are related to detoxification processes (Mueller et al., 2008). The most relevant OPDA-induced genes encode GSTs, cytochrome P450s and UDP-glucosyltransferases, and various transporters. Detoxification genes constitute the first line of defense against different stresses, so it is not surprising that they are induced by priming agents like Hx.

It is worth mentioning the similarity of the target genes activated in tomato in Hx-IR against *Botrytis cinerea* to those activated in *Arabidopsis* in menadione-IR against *Pst.* Genes like *GST*s, *WRKY18* and *WRKY40* can modulate the plant response in accordance with the challenging pathogen’s lifestyle. This can explain why the mode of action of priming agents is determined eventually by hosts and by the nature of the stress challenging them (Ahn et al., 2007). This evidence suggests that the regulation of the strategic components of plant signaling crosstalk is a key target of naturally priming agents, probably through the induction of epigenetic changes like histone acetylation. Further research using Hx as a model, and with other natural inducers, will elucidate the nature of these putative epigenetic changes.

**FIGURE 4:**
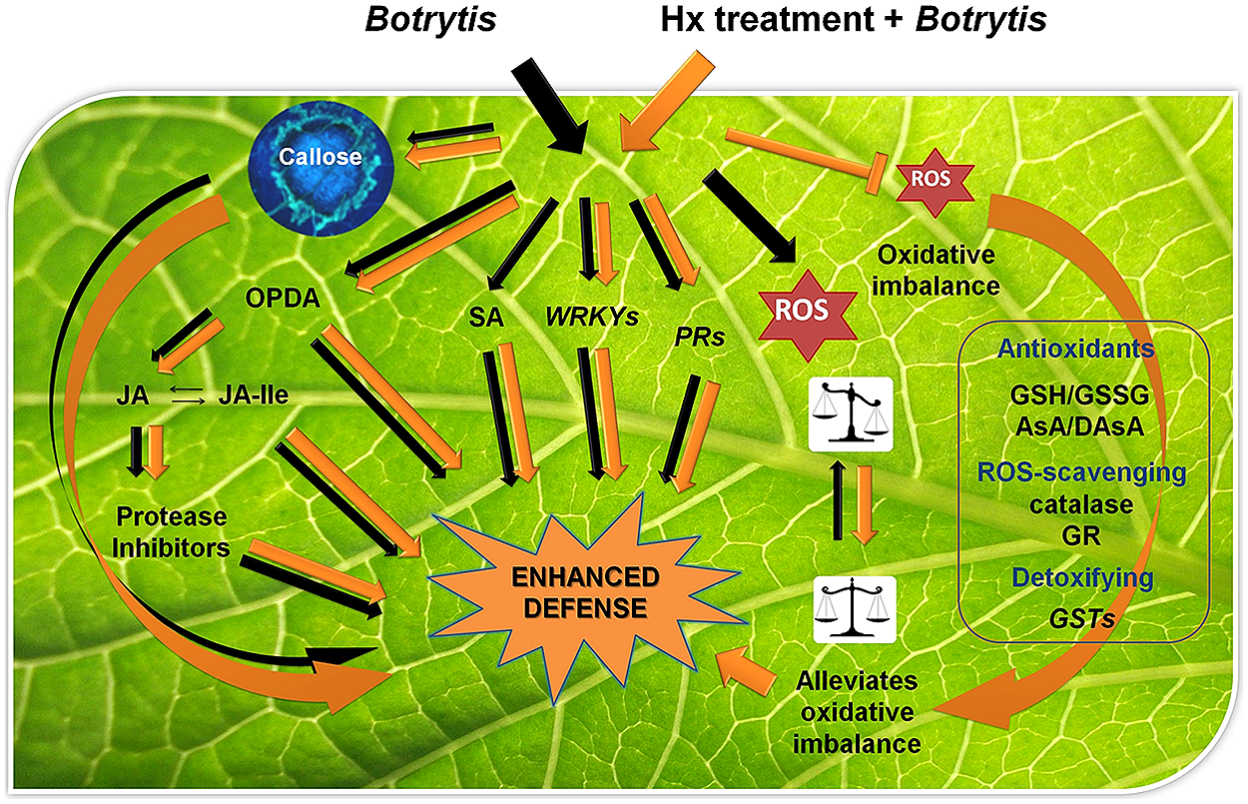
**Model for the Hx priming effect on plant defense mechanisms against *Botrytis cinerea*.** Black arrows indicate responses in untreated plants upon *Botrytis* infection. Orange arrows indicate induced responses in Hx-treated plants upon *Botrytis* infection. Hx-treatment increases *Botrytis*-induced responses enhancing callose, OPDA, JA and JA-Ile accumulation; potentiating transcript accumulation of genes like *WRKYs*, protease inhibitors and *PRs*, and inducing anti-oxidant, ROS-scavenging and detoxifying mechanisms. Hx, by counteracting the massive ROS accumulation induced by the fungus, alleviates the oxidative imbalance associated with *Botrytis* infection. Abbreviations: JA-Ile, jasmonoyl-isoleucine; GSH/GSSG, reduced/oxidized glutathione ratio; AsA/DAsA, reduced/oxidized ascorbate ratio; GR, glutathione reductase; GSTs, glutathione-S-transferases.

We propose Hx, a potent natural priming agent in a wide range of host plants and pathogens, as a model tool in this field of research (**Figure 4**). It can early activate broad-spectrum defenses by inducing callose deposition, in addition to the SA and JA pathways. Later it can prime pathogen-specific responses in each particular case according to the pathogen and its lifestyle. Interestingly, Hx primes redox-related genes and has an anti-oxidant protective effect, which might be critical for limiting the infection of necrotrophs.

## Conflict of Interest Statement

The authors declare that the research was conducted in the absence of any commercial or financial relationships that could be construed as a potential conflict of interest.
